# Metabolic profiling reveals reprogramming of lipid metabolic pathways in treatment of polycystic ovary syndrome with 3‐iodothyronamine

**DOI:** 10.14814/phy2.13097

**Published:** 2017-01-13

**Authors:** Ebru S. Selen Alpergin, Zeinab Bolandnazar, Martina Sabatini, Michael Rogowski, Grazia Chiellini, Riccardo Zucchi, Fariba M. Assadi‐Porter

**Affiliations:** ^1^ Department of Biological ChemistryJohns Hopkins UniversityBaltimoreMaryland; ^2^ Department of ZoologyUniversity of Wisconsin‐MadisonMadisonWisconsin; ^3^ Dipartimento di Patologia Chirurgica, Medica, Molecolare e Area CriticaUniversità di PisaPisaItaly; ^4^ Department of MedicineUniversity of Alabama BirminghamBirminghamAlabama; ^5^ Magnetic Resonance Facility at MadisonMadisonWisconsin

**Keywords:** 3‐iodothyronamine (T1AM), endocrine, lipid metabolism, metabolomics, Nuclear magnetic resonance (NMR) spectroscopy, polycystic ovary syndrome (PCOS), steroidogenesis

## Abstract

Complex diseases such as polycystic ovary syndrome (PCOS) are associated with intricate pathophysiological, hormonal, and metabolic feedbacks that make their early diagnosis challenging, thus increasing the prevalence risks for obesity, cardiovascular, and fatty liver diseases. To explore the crosstalk between endocrine and lipid metabolic pathways, we administered 3‐iodothyronamine (T1AM), a natural analog of thyroid hormone, in a mouse model of PCOS and analyzed plasma and tissue extracts using multidisciplinary omics and biochemical approaches. T1AM administration induces a profound tissue‐specific antilipogenic effect in liver and muscle by lowering gene expression of key regulators of lipid metabolism, PTP1B and PLIN2, significantly increasing metabolites (glucogenic, amino acids, carnitine, and citrate) levels, while enhancing protection against oxidative stress. In contrast, T1AM has an opposing effect on the regulation of estrogenic pathways in the ovary by upregulating STAR *,* CYP11A1, and CYP17A1. Biochemical measurements provide further evidence of significant reduction in liver cholesterol and triglycerides in post‐T1AM treatment. Our results shed light onto tissue‐specific metabolic vs. hormonal pathway interactions, thus illuminating the intricacies within the pathophysiology of PCOS. This study opens up new avenues to design drugs for targeted therapeutics to improve quality of life in complex metabolic diseases.

## Introduction

Polycystic ovary syndrome (PCOS) is a complex disease with endocrine and metabolic disorders. PCOS affects 10–20% of women in reproductive age with serious health outcomes such as infertility (Knochenhauer et al. 1998; Diamanti‐Kandarakis et al. 1999, 2004), obesity, type 2 diabetes, hyperandrogenism, and dyslipidemia (Cascella et al. 2008; Giallauria et al. 2008; Vinaixa et al. 2011). PCOS etiology is still unknown. However, environmental, (epi)genetic, and hormonal influences are thought to be important in the development of PCOS (Coles et al. 2013). Recently, we showed that PCOS women exhibit significantly diminished lipid oxidation, and perturbed glucose and amino acid metabolism (Whigham et al. 2014).

To understand the metabolic changes in PCOS, we developed a PCOS mouse model based on the method of Chapman et al. (2009) (Haviland et al. 2012). In this mouse model, we used prenatal glucocorticoid (GC) exposure that resulted in metabolic dysfunctions in adult females (Haviland et al. 2013). While GC treatment had milder inhibitory effects on the reproductive system, it still presented hallmarks of metabolic dysfunction in the adult female offspring consistent with those observed in women with PCOS (Haviland et al. 2012; Selen et al. 2015). Nuclear magnetic resonance (NMR)‐based metabolomics combined with breath ^13^CO_2_/^12^CO_2_ stable isotope fractionation analysis of GC‐mice showed a significant increase in fluxes through major pathways (pentose phosphate (PP) and tricarboxylic acid (TCA) energy metabolism), pointing to a higher NADPH level (Haviland et al. 2012) that leads to increased lipid synthesis. We hypothesized that preprogramming of the metabolic dysfunction in the female offspring results from increased fatty acids and cholesterol as precursors for steroid hormone synthesis. To test this hypothesis, we used 3‐iodothyronamine (T1AM) as anti‐hyperlipidemic agent.

T1AM is an endogenous compound structurally similar to the thyroid (T4) hormone but with distinct functional properties (Ghelardoni et al. 2014). A single dose of T1AM has opposing physiological effects (e.g., reduction in heart rate, metabolic rate, and body temperature) compared with thyroid hormone (Chiellini et al. 2007; Scanlan 2011). Previously, in an obese mouse model, we showed that single or chronic treatment with low‐dose T1AM (10 mg/kg/day) reduced body weight by 10–14% while maintaining glucose homeostasis (Braulke et al. 2008; Haviland et al. 2013). Furthermore, T1AM treatment resulted in increased lipid utilization without affecting food consumption (Haviland et al. 2013), making it a great candidate for treating metabolic dysfunction in GC‐mice that exhibited simultaneous increased lipogenesis and decreased lipid oxidation. In this study, we examined systemic changes and the tissue‐specific effects of subchronic T1AM treatment in the perturbed metabolic pathways and their associated gene signaling that affects lipid, cholesterol, and steroidogenic (hormonal) pathways in the GC‐mouse model.

## Materials and Methods

### Reagents

6‐Beta‐hydroxycortisol (Sigma‐Aldrich Corp., St Louis, MO), purified crystalline T1AM, was prepared as previously described (Scanlan et al. 2004). A vehicle solution was made using dimethyl sulfoxide (DMSO) and 0.9% medical grade saline (Hospira Corp. Lake Forest, IL).

### Animal study

All animal procedures and methods were carried out in accordance with the approved guidelines from University of Wisconsin, College of Letters and Sciences, Animal Care and Use Committee (Madison, WI protocol # L00408). We used outbred CD‐1 (*n* = 11) breeding pairs of mice from Harlan (Indianapolis, IN). All CD‐1 mice were acclimated for 1 week prior to mating and after a vaginal plug confirmed mating (gestational day, GD, 1). To generate PCOS mice, dams were assigned subcutaneous injections on GD 16 through GD 19 as previously described (Haviland et al. 2012). After weaning (~9 weeks), prenatally glucocorticoid‐treated F1 generation mice (GC‐mice) were fed AIN‐93G diet (17.7% protein, 60.1% carbohydrate, and 7.2% fat) (Haviland et al. 2012) until they reached about 50 g body weight at approximately 28 weeks of age. The adult female GC‐mice were then randomly chosen from different litters and assigned to two groups (*n* = 4): (1) Control GC‐mice (referred to as “GC”) were injected with the vehicle solution composed of certified organic sesame oil as a fat soluble liquid to minimize the amount of dimethyl sulfoxide (50 *μ*L), and (2) GC‐mice were treated with 25 mg/kg/day T1AM in the vehicle solution (GC+T1AM). Animals were fasted for 4 h each day prior to receiving T1AM treatment for a total of 5 days. Blood was collected on day ‐4 (before the treatment), and post‐treatment at day 5. At the end of the treatment period (day 5), mice were euthanized followed by immediate blood withdrawal and removal of tissues (skeletal muscle, liver, ovary, and subcutaneous adipose). Plasma was separated by heparinized tubes (Haviland et al. 2012; Selen et al. 2015). All tissues were stored at −80°C until further analysis.

### Sample preparation

#### Plasma

Plasma samples were processed for NMR experiments as previously described (Haviland et al. 2012). The samples were then reconstituted in plasma NMR buffer (i.e., 20 mmol/L phosphate buffer containing 1 mmol/L formate, 0.5 mmol/L 4,4‐dimethyl 4‐silapentane‐1‐sulfonic acid (DSS), and 0.1 mmol/L sodium fluoride (NaF)) and adjusted to pH 7.4 ± 0.05.

#### Tissues

About 50 mg of frozen tissue samples was homogenized in 10 mmol/L phosphate buffer (Selen et al. 2015). The dried supernatant was reconstituted in the tissue NMR buffer (i.e., D2O (99.9% ^2^H), containing 1 mmol/L formate, 0.5 mmol/L DSS, and 0.1 mmol/L NaF) and pH= 7.4 ± 0.05.

### Data Collection and Analysis

#### Metabolomics study

All 1D ^1^H‐NMR spectra were collected at 25°C on a 600‐MHz Varian VNMRS spectrometer as previously described (Haviland et al. 2012; Selen et al. 2015). 1D ^1^H‐NMR spectra were referenced to DSS (internal reference) and their concentrations were quantified relative to formate (1 mmol/L) by Chenomx software version 6 (http://www.chenomx.com). All data were log_2_‐transformed prior to analysis. Changes in the plasma and tissue metabolome of the T1AM treatment GC (GC+T1AM) and GC groups were evaluated using a partial least square discriminant analysis (PLSDA) (Lindgren et al. 1996; Pattini et al. 2008; Teng 2013) (www.metaboanalyst.com). In PLSDA, the variable on the projection (VIP) analysis shows metabolites ranked according to their influence on group separation (Eriksson et al. 2006). A subset of metabolites with higher VIP values in Table S1 were denoted with the letter “a” in Figure 2. Statistical significance was determined by Student's t‐test. A *P < 0.05 was set to significant and **P < 0.1 was considered moderately significant, where *n* = 4. Data are presented as mean ± standard error measurement (SEM). Heatmaps were generated based on the fold differences (FD) between GC+T1AM and GC groups as defined in equation 1 using R statistical software program (http://www.R-project.org). FD is defined by subtraction of a given metabolite concentration in the GC group, [metabolite]_GC_, from the corresponding metabolite concentration in the T1AM treatment group, [metabolite] _GC+T1AM_.

(1) $$\text{FD}={\left[\text{Metabolite}\right]}_{\mathrm{GC}+T1\mathrm{AM}}-{\left[\text{Metabolite}\right]}_{\mathrm{GC}}$$

#### Gene expression study

A total of 51 genes were profiled in four tissues (liver, skeletal muscle, adipose, and ovary) by real‐time RT‐PCR as previously described (Selen et al. 2015). Primers were designed in‐house using Beacon Designer Software v.7.9 (Premier Biosoft International, Palo Alto, CA) with a junction primer strategy (Table S2). Tissue samples were kept in RNALater‐ICE prior to processing. Total RNA was isolated with RNeasy Lipid Tissue Mini kit (Qiagen GmbH, Hilden, GER). Tissues (50–100 mg) were homogenized in QiaZol buffer (Qiagen) with TissueRuptor (Qiagen) for 30–40 s. RNA integrity, concentration, and purity were evaluated using Experion (Bio‐Rad Laboratories, Hercules, CA), and Cytation (Cytation 3, Winooski, VT), respectively. RNA was retrotranscribed using iScript cDNA Synthesis Kit (Bio‐Rad Laboratories, Hercules, CA). Relative quantification of gene transcripts was quantified using SYBR Green using RT‐PCR (Bio‐Rad Laboratories, Hercules, CA). For data normalization, reference genes from a list of tested genes (ACTB, RPLL13A, B2M, GUSB, HPRT, KDM2B, PPIA, PSMD4, and TBP) were chosen for muscle, ovary, and adipose tissues. Choice of reference genes was based on testing expression stability of nine candidate reference genes. RPL13A was selected as the best suitable reference gene to normalize liver RT‐PCR products under our experimental condition. All reactions were run in duplicate. Data were log_2_‐transformed prior to final analysis by the 2‐ΔΔCt method, as described by Livak and Schmittgen (2001). Statistical significance was determined by Student's t‐test. A P < 0.05 was set to significant and denoted with “*”. Data are presented as mean standard error measurement (SEM).

### Biochemical Analysis

#### Triglyceride (TG), cholesterol, and hepatic protein measurements

Liver samples were prepared for total triglyceride detection and quantification according to the manufacturer's manual (BioVision, Milpitas, CA). Liver total cholesterol (TC) was measured using colorimetric kit (Wako Diagnostics, Richmond, VA). Frozen liver samples (~10–20 mg) were homogenized in RIPA buffer (ab156034, Abcam, Cambridge, MA) that contained a protease inhibitor cocktail tablet (Roche Diagnosis, Mannheim, GR), and protein concentrations were quantified by Bradford assay (#500‐0205, Bio‐Rad Laboratories, Hercules, CA).

#### Plasma insulin, leptin, and adiponektin measurements

Plasma samples were prepared at the time of sacrifice as previously described (Haviland et al. 2012). Plasma insulin and leptin levels were measured using mouse ELISA kits (Crystal Chem, Downers Grove, IL). Adiponektin level was measured by adiponectin EIA kit (ACRP30, Norcross, GA).

## Results

### T1AM treatment results in weight loss and significant decreases in triglyceride and cholesterol levels

We administrated GC‐mice with T1AM (25 mg Kg^−1^ day^−1^) for 5 days to examine the effect of the drug on body weight regulation and lipid profiles. The T1AM treatment decreased body weight in GC‐mice by 14% compared with the untreated corresponding group (Table 1). In addition, T1AM reduced hepatic triglyceride (TG) and cholesterol (TC) levels (Table 1), without changing total hepatic protein level. Plasma glucose concentrations in GC‐mice are higher than in normal obese mice (data not shown). After T1AM treatment, glucose level was improved although it did not reach the level in normal obese mice.

**Table 1:** Biochemical parameters and body weight before and after 5 days of T1AM (25 mg/kg) administration

Measurements (units)	Group	Mean ± SEM
Blood Glucose (mg/dL)	GC	198.46 ± 10.51
	GC+T1AM	172.73 ± 26.01
Liver Total Triglyceride (mg/dL)	GC	2.294 ± 0.06
	GC+T1AM	1.268 ± 0.006^a^
Liver Total Cholesterol (mg/dL)	GC	50.98 ± 1.036
	GC+T1AM	34.00 ± 4.106^a^
Hepatic Protein Content	GC	76.73 ± 0.61
	GC+T1AM	77.25 ± 0.24
Blood Insulin (ng/mL)	GC	0.11 ± 0.02
	GC+T1AM	0.18 ± 0.02
Plasma Adiponectin (μg/mL)	GC	76.40 ± 41.72
	GC+T1AM	47.10 ± 41.19
Plasma Leptin (ng/mL)	GC	19.94 ± 2.65
	GC+T1AM	21.24 ± 3.19
Body weight (gr)	GC	49.425 ± 2.81
	GC+T1AM	42.450 ± 3.16

SEM, standard error measurement.

Data are shown as standard mean values ± SEM.

a Shows statistical significance level by student‐test (*P* < 0.05 (*n* = 3)).

We examined the effect of T1AM on hormones regulating glucose and lipids. Trends for decreased insulin levels mirrors lowered glucose level in GC‐mice. Plasma adiponectin level showed a decreasing trend while plasma leptin level increased in the T1AM treatment group (Table 1).

### Metabolome profiles of plasma, liver, and muscle in T1AM‐treated GC‐mice show normalization of major perturbed metabolic pathways

**Figure 1:**
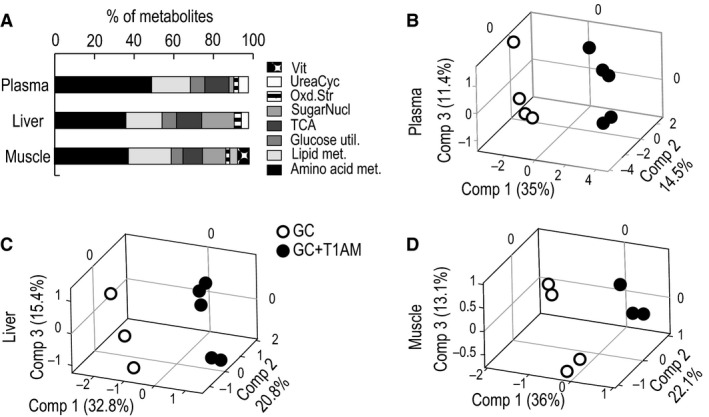
Multivariate analysis of plasma, liver, and muscle tissues. Panel A shows distribution of profiled metabolites into major pathways in plasma, liver, and muscle tissues, respectively. Panels B, C, and D represent partial least square discriminant analysis (PLSDA) score plots of plasma, liver, and muscle metabolomes, respectively. White circles represent GC‐mice, and black circles represent GC+T1AM treatment group. Vit, vitamin metabolism; UreaCyc, urea cycle; Oxd.Str, oxidative stress; SugarNucl., sugar nucleotide metabolism; TCA, citric acid cycle; Glucose util., glucose utilization – glycolysis; Lipid Met., lipid metabolism; Amino acid met., amino acid substrates.

**Figure 2:**
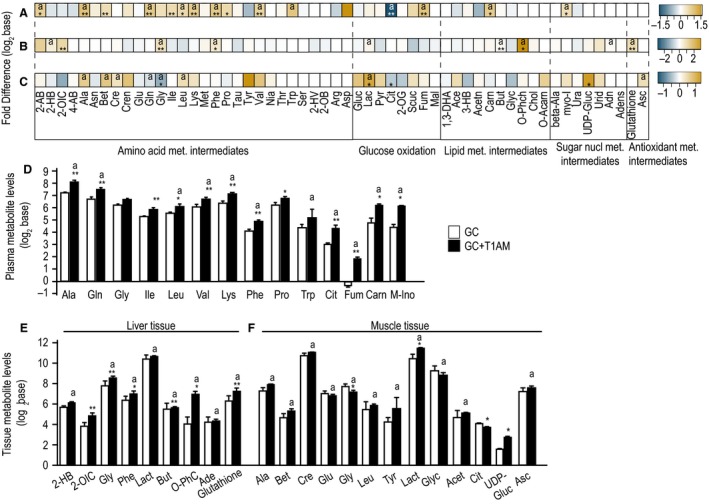
Heatmap of metabolite levels in response to T1AM treatment. Differential changes (GC+T1AM – GC) for a given metabolite in (A) plasma**,** (B) liver, and **(**C) muscle. Metabolome profiles are color‐coded for each tissue, gold and shades show >0, and blue and shades shows< 0, and values are in log_2_ base. (D–F) show significantly changed (*P* < 0.05 or *P* < 0.1, denoted by “**” or “*,” respectively) metabolites along with the metabolites identified as “important” by partial least square discriminant analysis (PLSDA) method (denoted by “a”). *X*‐axis shows metabolites' ID, and *y*‐axis shows concentration levels in log_2_ base, GC‐mice (white bar), T1AM‐treated GC‐mice (GC+T1AM, black bar). ** *P* < 0.05; * *P* < 0.1, Student's t‐test.

Metabolic profiling of plasma, muscle, and liver tissues using ^1^H‐NMR analysis identified 60 metabolites that belong to major pathways (Fig. 1A). PLSDA analysis of plasma, liver, and muscle metabolome profiles shows clear separations between GC+T1AM‐treated and GC groups in each respective tissue (Fig. 1B–D, and Table S1) indicating marked effects of T1AM treatment, which mirrors the biochemical phenotype associated with weight loss **(**Table 1). To visualize the magnitude of change in the metabolome, we applied fold difference (FD, equation 1 in Methods) to the metabolites in each tissue with outputs shown in heatmap representations (Fig. 2A–C). Our results show that while some metabolic changes are common among all tissues such as in liver, muscle, and plasma, there are a number of tissue‐specific metabolic changes in the GC+T1AM‐treated group compared with the GC group.

#### Plasma

Decreased citrate and amino acids levels were previously observed in women with PCOS (Atiomo and Daykin 2012; Zhao et al. 2012; Whigham et al. 2014). We compared fold differences of metabolites in plasma of GC+T1AM‐treated and control GC‐mice with metabolites (*P* < 0.1) that are altered (Fig. 2A). These metabolites including major increases in TCA cycle intermediates citrate (Cit) and fumarate (Fum), amino acids and intermediates (2‐aminobutyrate (2‐AB), alanine (Ala), betaine (Bet), glutamine (Gln), isoleucine (Ile), leucine (Leu), lysine (Lys), phenylalanine (Phe), proline (Pro), valine (Val)), in addition to an important fatty acid carrier, carnitine (Carn), a sugar nucleotide metabolism intermediate, myo‐inositol (myo‐I) were all increased after T1AM treatment (Fig. 2D). Direction of changes particularly in amino acids and citrate levels indicates “normalization” as the major metabolic effect of T1AM on altered pathways is consistent with previously found biomarkers in women with PCOS.

#### Liver

Consistent with plasma data, T1AM treatment altered hepatic amino acid metabolism pathway intermediates (2‐hydroxybutyrate (2‐HB), 2‐oxoisocaproate (2‐OIC), phe, and gly). Levels of glutathione, the substrate in a major nonenzymatic antioxidant pathway, increased significantly indicating a greater redox level due to T1AM treatment (Fig. 2C).

#### Muscle

Metabolites that have important influences on the group separation of metabolome profiles are shown in Figure 2C and F. Levels of amino acid metabolic intermediates, glucose oxidation intermediates, lipid metabolism intermediates (acetate), PP pathway intermediate (UDP‐glucose, UDP‐G), and a nonenzymatic antioxidant metabolism intermediate ascorbate (Asc) were increased, while glycerol (glyc), cit, and glycine (gly) were all decreased after the treatment.

### Tissue‐specific targeted gene transcription analysis by RT‐PCR

**Figure 3:**
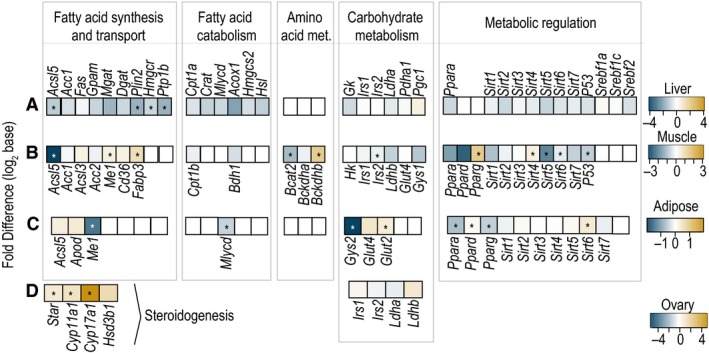
Heatmap representation of fold difference of expressed genes profiled by RT‐PCR. Differential changes in gene expression (GC+T1AM – GC) are color‐coded in panels A–D for liver and muscle, adipose and ovary, respectively. Gold and shades show > 0, and blue and shades show < 0 in log_2_ base. * *P* < 0.1, Student's t‐test.

To correlate changes in metabolic profiles, we examined changes in gene transcription for targeted metabolic pathways in carbohydrate metabolism, fatty acid synthesis, catabolism and transport, glucose, and steroidogenic pathways in several tissues (Table S2). Figure 3A–D shows a heatmap of fold difference in gene expression levels in liver, skeletal muscle, adipose, and ovary tissues after GC+T1AM treatment compared with the GC group. After T1AM administration, most genes in lipid and carbohydrate metabolism were all downregulated consistent with metabolic profiles with the exception of steroidogenic pathways.

### T1AM treatment shows differential tissue‐specific effects on lipid and cholesterol metabolic pathways

The T1AM treatment had tissue‐specific effects on fatty acid metabolism and signaling pathways in the GC‐mouse model. In particular, T1AM had general effects on major metabolic pathways (lipid, carbohydrate, and amino acid) in muscle and adipose tissues, whereas in liver, it mainly decreased gene transcription levels in lipid and cholesterol metabolism (Fig. 3A–D).

#### Liver

**Figure 4:**
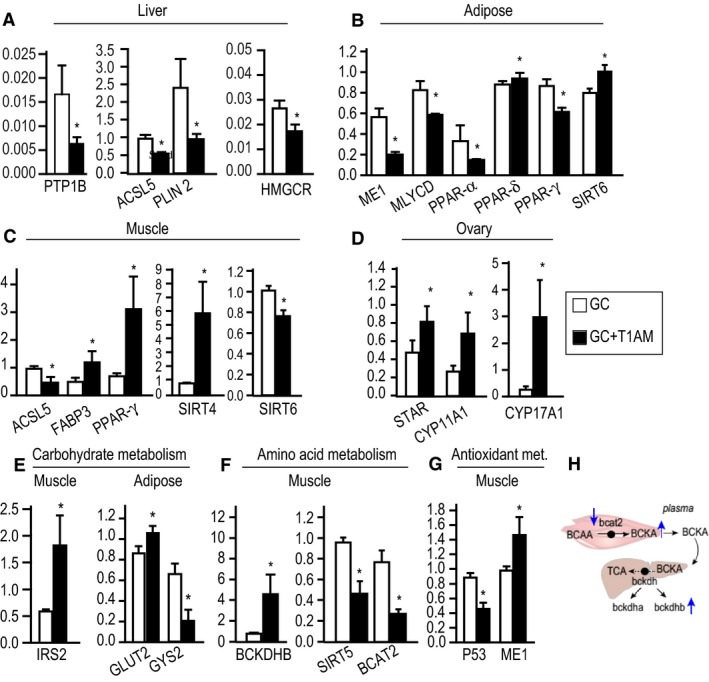
Changes in transcription levels in major metabolic pathways in T1AM‐treated GC‐mice. Panels A–G show lipids, carbohydrates, amino acids, and antioxidant pathways in liver, adipose, muscle, and ovary, respectively. In all graphs, black bars represent treatment and white bars represent control groups, *x*‐axis: gene IDs, and *y*‐axis: gene expression levels relative to RPL13A by RT‐PCR. * *P* < 0.1. Data are shown as mean ± standard error measurement (SEM). 4 h**.** Schematics of branch chain amino acid catabolism are shown in muscle and liver. Solid black arrows represent direction of the flow in the corresponding pathway, and dotted black arrows indicate one or more reactions in the corresponding pathway. Black circle indicates corresponding gene for each pathway. Up and down blue arrows show increased and decreased gene or metabolite levels. BCAA: branch chain amino acid; BCKA, branch chain keto acid; TCA, citric acid cycle; BCAT2, branch chain acetyltransferase 2; BCKDHa and BCKDHb, branch chain keto acid dehydrogenase subunits a and b.

Four major hepatic genes were downregulated by T1AM treatment. These key genes are as follows: (1) protein tyrosine phosphatase 1B (PTP1B) that plays a major role in the intersection of insulin and lipid regulatory signaling pathways, (2) perilipin 2 (PLIN2) that regulates storage and hydrolysis of neutral lipids, (3) acyl‐CoA synthetase long‐chain family member 5 (ACSL5) that has a key role in FA activation, and (4) 3‐hydroxy‐3‐methylglutaryl‐CoA reductase (HMGCR) that is a key enzyme in the initial step of cholesterol synthesis (Fig. 4A).

Additional hepatic genes showed only decreasing trends. These are ACC1, in de novo lipogenesis (DNL); FAS, in FA synthase; glycerol‐3‐phosphate acyltransferase (GPAM), monoacylglycerol transferase (MGAT), and diacylglycerol transferase (DGAT), in triglycerides (TG) synthesis; and regulator (SREBP2), in cholesterogenesis (Fig. 3A).

Further support for the harmonic effect of T1AM treatment on acute hepatic lipid metabolism pathways comes from attenuation in expression levels of several regulatory genes (PPAR‐α*,* sirtuins (SIRT3, SIRT5, and SIRT6)) for FA catabolism and their target pathways (malonyl‐CoA decarboxylase (MLYCD*)*, acyl‐CoA oxidase 1 palmitoyl (ACOX1), 3‐hydroxy‐3‐methylglutaryl‐CoA synthase 2 (HMGCS2), and the mitochondrial FA transporter (carnitine palmitoyltransferase 1a and carnitine O‐acetyltransferase (CPT1A and CRAT)) (Fig. 3A, Table S2).

#### Skeletal muscle

Muscle lipid metabolism (utilization and synthesis) and the regulatory gene transcription levels (Fig. 3A and Fig. 4B) were distinct from liver. The concerted increased levels of three key genes were as follows: FABP3, a gene that encodes a protein for long‐chain FA transport and oxidation; PPAR‐γ, peroxisome proliferator‐activated receptor gamma that is a transcriptional regulator of lipogenic genes; and SIRT4 with an important regulatory role in beta‐oxidation in lipid metabolism. On the other hand, T1AM also resulted in harmonic downregulation of ACSL5, and SIRT6 consistent with the notion of increased oxidation of long‐chain FAs and possibly their turnover in the muscle.

A number of genes associated with FA synthesis (ACC1, ACC2, ACSL3, CD36*)* and FA oxidation (CPT1b and BDH1 (Table S2) showed decreasing levels, which are consistent with observed major changes in lipid metabolic pathways through T1AM treatment (Figs. 3B and 4C). Interestingly, a metabolic regulator (SIRT5), and tumor suppressor p53 (P53, an important mediator of metabolic and mitogenic pathways) were reduced after the treatment with T1AM.

#### Adipose

T1AM induced specific changes in expression of six genes that regulate lipid metabolism in white adipose tissue (Fig. 4B). The ME1 gene decreased by ~ threefold, which encodes malate dehydrogenase enzyme with antioxidant metabolic activity and produces NADPH for lipid synthesis. The expression level of adipogenic regulatory gene, PPAR‐γ, was also reduced in the T1AM treatment group. Expression levels of the positive transcriptional regulator of genes involved in lipid catabolism (*PPAR‐α*) and one of its targets, MYLCAD*,* which catalyzes the production of acetyl‐CoA from malonyl‐CoA, were downregulated in treated GC‐mice (Fig. 4B). On the other hand, the decreased gene expression level of SIRT6, a long‐chain fatty acid binding protein, suggests increased lipolysis and release of fatty acids from the adipose tissue.

ACSL5 and APOD (encodes apolipoprotein) transcription levels in the T1AM‐treated group showed increasing trends in fold change analysis (Fig. 3C), which are consistent with the overall observed effect of T1AM on increased lipid catabolism and decreased lipogenesis in adipose tissue.

#### Ovary

To understand the effect of T1AM on the cholesterol metabolism in the reproductive tissue, we examined expression levels of several genes in steroidogenic pathways in ovary. Remarkably, T1AM treatment significantly increased expression of key genes in steroid and cholesterol metabolism (Fig. 3D) in this tissue. T1AM‐treated mice elevated STAR transcript that encodes for steroidogenic acute regulatory protein and coordinates cholesterol uptake and its movement into mitochondria, CYP11a (also known as P450SCC) gene that encodes for cholesterol side chain cleavage enzyme and catalyzes the rate‐limiting step of steroidogenesis (i.e., the conversion of cholesterol to pregnenolone (Miller 1988)), and CYP17A1 that encodes steroid 17‐alpha‐monooxygenase enzyme and participates in androgen synthesis (Fig. 4D). These changes indicate that response to T1AM in the ovary of GC‐mice is different and more likely due to its major pathophysiology as compared to other metabolic tissues.

### T1AM alters the biomarkers associated with glucose metabolism pathways

Previous data suggested that glucose metabolism was changed in both nondiabetic GC‐mice and women with PCOS. To further examine effect of T1AM on regulating glucose metabolism in GC‐mice, we measured transcriptional levels of key signaling (PTP1B), regulatory (GLUT2), transport (IRS2), and storage (GYS1) genes in glucose metabolism. Our gene expression data indicate that T1AM significantly changes expression of a few key genes in carbohydrate metabolism in liver, muscle, and adipose tissues.

In the liver, expression of PTP1B that encodes a protein in the insulin signaling pathway was reduced by T1AM treatment, suggesting a normalization of glucose metabolism in hepatic tissue of GC‐mice (Fig. 4A). In muscle tissue, IRS2 gene expression was significantly upregulated in the T1AM treatment group (Fig. 4E). In adipose tissue, GLUT2 that encodes glucose transporter 2 protein was increased, whereas the GYS1 gene that encodes glycogen synthase enzyme (in the rate‐limiting step in glycogen synthesis) was downregulated in T1AM‐treated GC‐mice (Fig. 4E), suggesting a shift from glucose to lipid metabolism. Changes in gene expression in muscle mirror those changes seen in metabolome profiles that show increasing level of branched amino acids and decreased level of glycine pointing to increased insulin sensitivity in T1AM treatment.

### T1AM treatment alters gene expression levels of the branched chain amino acids in muscle

To confirm observed changes in metabolic profiles of branched chain amino acids (BCAA) in skeletal muscles, we analyzed transcription levels of genes associated with transamination and catabolism of BCAA metabolism. Our results indicate that these genes were significantly altered by T1AM treatment in muscle. The gene for branched chain amino acid transaminase 2 (BCAT2) that degrades BCAAs into branched chain keto acids (BCKA) decreased, whereas expression of branched chain keto acid dehydrogenase beta subunit (BCKDHB) gene level increased in T1AM‐treated GC‐mice (Fig. 4F and H). Coincidently, an important metabolic regulator of ammonia detoxification and disposal (SIRT5) expression level was significantly downregulated in the T1AM‐treated group (Fig. 4F).

### T1AM treatment increases antioxidant pathways in muscle and liver

The metabolic profiling showed increased level of antioxidant in tissues after T1AM treatment. We examined a number of genes in oxidative stress pathways. P53 gene expression level is generally lower in healthy cells. However, oxidative stress (Olovnikov et al. 2009) and activated oncogenes (Harris and Levine 2005) are known to induce expression levels of the P53 gene. In muscle, P53 transcription level was reduced after T1AM administration in the GC‐mice (Fig. 4G, *P* < 0.1). Furthermore, the gene expression level of ME1 was upregulated in muscles from the treatment group compared with GC‐mice (Fig. 4G). Increased gene levels in antioxidant metabolism are consistent with an observed increased level of ascorbate (Fig. 2F) in muscle.

In liver, P53 expression level also showed a decreasing trend in the T1AM treatment group (Fig. 3A), consistent with the observed increased level of glutathione (Fig. 2E).

## Discussion

Increased lipogenesis, TG, and cholesterol levels have been associated with complex metabolic disorders in women with PCOS (Whigham et al. 2013) and in animal models (e.g., rhesus monkey (Abbott et al. 2009) and prenatally treated GC‐mice (Haviland et al. 2012) leading to abnormalities and associated risk factors in developing lipotoxicity (Lelliott and Vidal‐Puig 2004), fatty liver, type 2 diabetes (T2D), infertility (Moran et al. 2015), and cardiovascular diseases (CVD)(Group, 2004; Diamanti‐Kandarakis et al. 2007).

The GC mouse is a suitable corresponding model for metabolic impairments in PCOS (Haviland et al. 2012). Previously, we showed 5 days of treatment with low‐dose T1AM was optimum for weight reduction in normal obese mice without affecting muscle breakdown (Haviland et al. 2013). Our main aim in this study was to examine whether T1AM treatment could normalize metabolic dysfunction in GC‐mice and verify those metabolic changes by targeted gene transcription and biochemical assays.

**Figure 5:**
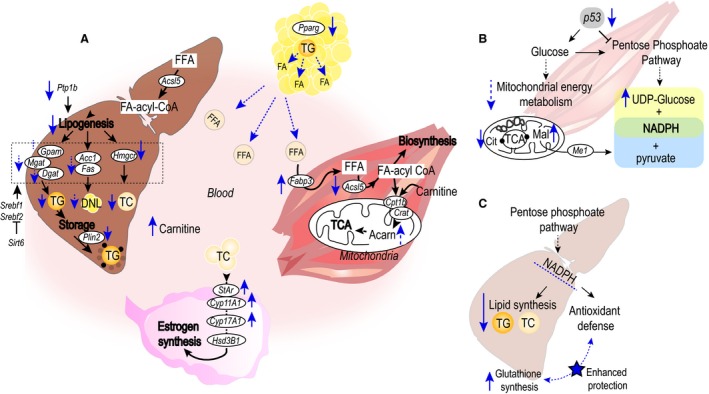
Schematics of multitargeted actions of T1AM on metabolic and steroidogenic pathways. (A) Schematics of lipid metabolites and gene pathways in major tissues. The T1AM treatment group results in release of lipids from adipose tissue and consumption by muscle and liver while decreases lipid and cholesterol synthesis, and lipid storage in liver. However, T1AM results in increased estrogen synthesis in ovaries. (B) A schematic of T1AM action on glucose metabolism in muscle. T1AM normalizes glucose metabolism away from pentose phosphate pathway and lipid synthesis (by downregulation in expression of the P53 gene, and increased UDP‐glucose, pyruvate, and NADPH metabolites) toward glucose utilization in the mitochondria energy metabolism that leads to increased malate dehydrogenase (ME1) gene expression; (C) A schematic of the proposed T1AM effects on lipid and antioxidant pathways in liver. T1AM increases the hepatic antioxidant defense mechanism shown by glutathione accumulation. Solid arrows represent the direction of flow in the corresponding pathway, and dotted black arrows indicate one or more reactions in the corresponding pathway. Dotted arrow represents a trend toward the direction of the arrow. Abbreviations: Acarn: acetylcarnitine, Fatty acyl‐CoA: fatty acyl co‐enzyme A, FFA: free fatty acid, DNL: de novo lipogenesis.

The NMR metabolomics data show that subchronic treatment with a pharmacological dose (25 mg kg^−1 ^day^−1^) of T1AM induces multiple effects in GC‐mice that normalize energy metabolism, increase lipid and antioxidant metabolic pathways in a tissue‐specific manner. Figure 5 shows schematics of changes in metabolic and gene pathways in each tissue. Complementary targeted gene transcriptome profiles show specifically that T1AM downregulates lipid and cholesterol synthetic pathways in liver and muscle. Unlike changes seen in metabolically active tissues, liver and muscle, steroidogenic pathways are upregulated in ovary of GC‐mice pointing to the origin of dysregulation of these pathways. Although T1AM results in overlapping changes in regulatory and/or signaling genes in different tissues, the metabolic lipid metabolism pathways in liver are distinct from adipose and muscle, suggesting that T1AM may have a multitargeted cellular‐specific mechanism of action that need to be examined further.

In liver, T1AM specifically affects the cholesterol pathway and lipid storage and breakdown. Changes in hepatic metabolic lipid pathways are mapped to downregulation of two key genes, PTP1B and PLIN2 that play important roles in regulating lipogenesis, that is, TG storage and lipolysis (Fig. 5). Two other genes, ACSL5 and HMGCR, at the crossroad between FA activation and the rate‐limiting step in cholesterogenesis, are also downregulated. In addition, decreasing trends in other lipogenic genes in TG synthesis and DNL support overall observed improvements in the hepatic lipid and cholesterol profiles.

Increased levels of PPAR‐γ have been shown to regulate lipid metabolism by increasing fat storage and synthesis (Janani and Ranjitha Kumari 2015) in adipose tissue. Treatment with T1AM protects against lipogenesis in GC‐mice through significant downregulation of PPAR‐γ along with upregulation of SIRT6 transcription in adipose tissue (Figs. 4B and 5A). Increased expression level of SIRT6 inhibits lipogenic transcription factors SREBP1 and SREBP2 that regulate glycolysis and fatty acid metabolism, while it mediates cholesterol homeostasis by lowering LDL cholesterol under normal and HFD conditions (Kanfi et al. 2010). In addition, SIRT6 is shown to bind to long‐chain FAs (Feldman et al. 2013). Our data show an increase in SIRT6 transcription level (Fig. 4B) that further supports downregulation of DNL and cholesterogenesis possibly through increased release of long‐chain FAs from adipocytes and their clearance in muscles as a major energy source for FA oxidation by a reduction in ACSL level in muscle and liver (Fig. 4A and 4C).

We previously showed that a chronic treatment with the lowest pharmacological dose of T1AM (10 mg/kg) in normal obese mice induces lipid oxidation (Haviland et al. 2013) in addition to weight loss. Here, we show increased FA utilization in muscles through upregulation of FABP3 (Figs. 4A and 5A). FABP3 has been shown to increase in response to FA exposure in vitro and in vivo studies (Veerkamp and van Moerkerk 1993; Zanotti 1999). The fabp family of proteins functions in transporting long‐chain FAs to different cellular compartments, for example, mitochondria and peroxisomes for FA oxidation (Atshaves et al. 2010). In addition, acetylcarnitine is significantly increased in T1AM‐treated muscles from GC mice. Acetyl‐carnitine plays key roles during FA transport and the initial step of FA oxidation in mitochondria (Fritz and Mc 1959; Bremer 1962; McGarry and Brown 1997). Furthermore, the increased carnitine level in plasma supports a higher demand for fatty acid oxidation through the carnitine‐mediated pathway to match the increased lipid utilization level (Figs. 2D and 5A).

In muscle, both SIRT6 and P53 gene expression levels (Fig. 4C and G) are significantly decreased consistent with reduction of the citrate metabolite in the TCA cycle (Fig. 2F), leading to a “normalized” energy balance and a less oxidative environment. SIRT6 protein suppresses several genes through histone modifications, including glucokinase, pyruvate kinase, FA synthase, and acetyl‐CoA carboxylase (Kanfi et al. 2010). This is consistent with the current observations that suggest an alternative epigenetic regulatory mechanism by T1AM which requires further examination.

T1AM has an additional metabolic correction by increasing protective antioxidant defense mechanisms in key highly metabolic tissues (Fig. 5). Previously, we showed increased oxidative stress in kidneys from GC‐mice compared with normal mice under similar diets (Selen et al. 2015). In PCOS women, inefficient neutralization of ROS is reported to increase oxidative stress (Gonzalez et al. 2006; Victor et al. 2009) independent of their weight, age, or metabolic abnormalities. Glutathione and ascorbate are nonenzymatic antioxidant molecules that trap and neutralize ROS reactions at the expense of NADPH, as an essential cellular redox and buffering agent (Bendich et al. 1986; Meister 1992; Mari et al. 2009). Depletion of glutathione is shown during excess mitochondrial cholesterol loading in a mouse model (Mari et al. 2006), while reduction in ascorbate level is associated with pathological accumulation of cholesterol and TG in liver and other tissues (Nambisan and Kurup 1975; Holloway and Rivers 1981). In addition, a Ascorbic acid acts like an antioxidant that protects against lipid peroxidation (Choi et al. 2016), or functions as an indirect antioxidant, providing electrons to generate reactive forms of other antioxidants such as glutathione (Bourges et al. 2005). Taken together, increased levels of glutathione in liver and ascorbic acid in muscle indicate that the oxidative environment in the tissues are reduced despite increased lipid oxidation. T1AM also downregulates P53 in liver while upregulating ME1 in muscle, which are accompanied with increased levels of ascorbate, glutathione, and UDP‐glucose. These pathways could generate NADPH as another potential antioxidant molecule that is shared in intersecting pathways between lipogenesis and antioxidant metabolic reactions to lower the oxidative condition in tissues (liver and muscle).

T1AM treatment improves nitrogen and amino acid metabolism in muscles from GC‐mice by downregulating SIRT5*,* an important regulator of nitrogen metabolism, BCAT2 expression, and BCAA metabolite levels (Fig. 4F). We previously showed that nighttime nitrogen excretion in urine was positively correlated with circulating testosterone levels in PCOS women (Whigham et al. 2013). Previous studies showed that increased levels of BCAA and decreased level of Gly improve insulin sensitivity (Muoio et al. 2002). BCAA levels are increased while Gly is decreased in muscles from T1AM‐treated GC‐mice, indicating that insulin sensitivity is improved in nondiabetic GC‐mice.

In contrast to other tissues, T1AM has opposing effects on cholesterol metabolism leading to steroidogenesis in ovaries of GC‐mice (Fig. 5D). In the normal ovary, androgens are synthesized from cholesterol in the theca cells. The ovary is also the primary source of increased circulating androgens (Franks 1995; Legro et al. 1998; Ehrmann 2005). In the ovary from a subset of PCOS women (25–60%) (Moran et al. 1999; Kumar et al. 2005), increased adrenal steroidogenesis contributes to increased circulating androgen and impaired insulin signaling as phenotypic hallmarks of (Handwerger et al 2008), making it difficult to pinpoint which upstream gene networks and metabolic signaling pathways participate in the manifestation of these PCOS phenotypes. However, treatment of PCOS women with insulin‐sensitizing drugs (e.g., metformin, pioglitazone) results in a reversal of ovarian phenotype (Legro 2001). In our study, T1AM lowers fasting circulating glucose levels in GC‐mice (Table 1) but with trends in increasing insulin level, which suggest an upstream glucose–lipid signaling regulatory mechanism. The effect of subchronic application of low pharmacological dose of T1AM on cholesterol that leads to estrogenic hormone synthesis in ovaries of GC‐mice is the opposite of other metabolic tissues. This observation may be due to an increased supply of cholesterol and FAs as precursors for estrogenic hormone synthesis, nevertheless it points to fundamental dysregulation of hormonal pathways in the ovary of GC‐mice, which requires further examination.

Leptin and adiponectin are adipose tissue‐derived hormone peptides that play important regulatory roles in energy, glucose homeostasis, and lipid metabolism (Robinson et al. 2011). Despite decreased body weight and improved lipid profile, the circulating levels of hormones were not significantly reduced. This may be due to our lower animal number derived from diverse outbred CD‐1 genetic background or the shorter length of treatment period. In particular, in the anti‐obesity hormone, leptin level is not only positively correlated with body fat content, but also negatively correlated with lower energy intake and energy stores (Boden et al. 1996). Level of leptin in normal subjects is correlated with a number of endocrine hormones such as insulin, glucocorticoid, and testosterone (Janeckova 2001). However, the increased level of leptin after T1AM treatment may be due to the preexisting pathophysiology in our model of prenatal exposure to a glucocorticoid.

At present, direct intracellular mechanism of T1AM action is not known; multiple lines of research including this study support the hypothesis that T1AM is a multitargeted ligand. Although T1AM is structurally similar to TH hormone, functionally T1AM's action is through a different mechanism. In TH nuclear receptor‐binding assays and TR reporter gene activation assays, T1AM shows neither affinity for TRα or TRβ, nor an ability to stimulate or inhibit nuclear TR‐mediated transactivation (Scanlan et al. 2004). At present, the only known molecular targets of T1AM are trace amine receptor (TAAR1), α2A adrenergic receptor, DAT, and NET VMAT2 proteins (Snead et al. 2007). While T1AM inhibits monoamine transport, it is shown to be a potent agonist of the G protein‐coupled trace amine receptor TAAR1 (Scanlan et al. 2004). The presence of TAAR1 receptor in the brain may be in part responsible for the metabolic responses, such as changes in fuel utilization, food intake, and behavioral activity. At physiological level, T1AM is considered an endogenous hormone that when administered in rodents at high dose (50 mg/kg/day) depresses metabolism by a rapid interruption of carbohydrate fueling accompanied by a compensatory rise in lipid utilization (Braulke et al. 2008), and shift in metabolism from carbohydrate to lipid breakdown (Haviland et al. 2012). T1AM inhibits insulin secretion and induces hyperglycemia in mice via Gi signaling through the α2A receptor while its activation of the Gs‐coupled TAAR1 stimulates insulin secretion and results in hypoglycemia when the α2A receptor is blocked or absent (Ianculescu and Scanlan 2010). The targeted metabolomics research opens up a new avenue for future research to examine the new intracellular signaling mechanism of T1AM action through regulatory and/or signaling (e.g., sirtuin‐mediated) pathways.

In summary, our results reveal a new paradigm about the downstream effect of T1AM treatment in GC‐mice through gene signaling that regulates lipid and cholesterol metabolism differentially (Fig. 5). This mechanism of action is distinct from T1AM's acute rapid onset of metabolic effects mediated by cell surface G protein‐coupled receptors as previously proposed by Scanlan et al. (2004). Taken together, these findings provide a basis for understanding T1AM's increased lipolytic and decreased lipogenic metabolic effects. Thus, T1AM may serve as a promising endogenous supplement in the treatment of dysfunctional lipid metabolism such as fatty liver disease, obesity, and PCOS. This study could open up a new avenue for future research to understand alternative modes of hormone metabolic pathway‐mediated regulation and develop new approaches for essential therapeutic targets in order to manage complex metabolic diseases and improve health outcomes in women with PCOS.

## Conflict of Interest

The authors declare no competing financial interests.
